# Histological and Biochemical Assessment of Camel Milk in Protective and Therapeutic Aspirin‐Induced Gastric and Liver Damage in Rabbits

**DOI:** 10.1155/vmi/2850214

**Published:** 2026-07-02

**Authors:** Hayder Dawood Saleem, Zeid Muhsen Alsadoon, Ali Hussein Fadhil, Anfal Jabbar Taher

**Affiliations:** ^1^ Department of Medical Laboratory Techniques, Al-Manara College For Medical Sciences, University of Manara, Al-Amarah, Iraq, uomanara.edu.iq; ^2^ Department of Microbiology, College of Veterinary Medicine, University of Wasit, Wasit, Iraq, uowasit.edu.iq; ^3^ Department of Internal Medicine and Preventive, College of Veterinary Medicine, University of Kerbala, Karbala, Iraq, uokerbala.edu.iq; ^4^ Department of Medical Laboratory Techniques, University of Manara, Maysan, Iraq

**Keywords:** aspirin toxicity, camel milk, gastric ulceration, liver function, rabbits

## Abstract

The study aimed to investigate the potential protective effects of camel milk against aspirin‐induced gastric mucosal injury. The role of camel milk therapy in improving health status in rabbits was investigated through diagnostic and histological examinations of gastric tissues. All rabbits, 32 New Zealand white rabbits weighing between 1200 and 1800 g and aged between 3 and 5 months, were housed in standard laboratory environments (60% humidity, 20°C–25°C, and typical field rearing), and the animals were randomly divided into four groups, each containing eight rabbits. The control Group S represented the control group, while Group S1 received camel milk (15 mL/day) orally once daily. The Group S2 received a dose of 150 mg/kg body weight (BW) of the drug aspirin once daily oral dose. Whereas the Group S3 received a 15 mL/day dose of camel milk, Group S3 received camel milk 15 mL/day together with aspirin 150 mg/kg BW/day. The drug aspirin was administered once daily. The experiment lasted for a period of 70 days in a controlled environmental laboratory after performing a histopathological examination of the stomach tissues, as well as a biochemical analysis of liver function markers. The incidence and severity of gastric ulcers were significantly reduced in the camel milk–treated group compared to the aspirin group (*p* < 0.05). In addition, the significant differences (*p* < 0.01) in liver function were observed in variation in ALT, DB, TP, albumin, and ALP in Group S1 in comparison to Group S. The results show a nonsignificant decrease in AST and TB levels in both Groups S1 and S3, compared to Group S. The histological results also showed a decrease in inflammatory infiltration and tissue damage in the rabbit Group S3 compared to the Group S2 that consumed aspirin only. In conclusion, the results showed that one of the properties of camel milk, antioxidant and anti‐inflammatory activity, is highly effective in preventing and treating aspirin‐induced gastric and liver damage.

## 1. Introduction

The camel, as a desert animal, plays an important economic role in the dry desert and semidesert areas of Iraq for thousands of years. Camels have been used in the everyday life of some people, especially those living in remote areas and harsh conditions [1]. The camel is still a highly valued animal today because of its products, including milk, which is important in treating certain health problems [2]. Recently, camel milk has been used for a number of purposes in treating some human health problems, especially globally, due to its high therapeutic value [3]. However, it contains high levels of primary nutrients such as “Na, K, Fe, Zn, and Mg,” as well as high concentrations of β‐insulin‐like proteins, in addition to some important vitamins, particularly ascorbic acid, thiamine, and riboflavin, also relatively low cholesterol levels and contains high‐quality proteins [4, 5]. Camel milk is also characterized by antioxidant, antibacterial, antiviral, and antifungal activities due to the presence of bioactive compounds such as lactoferrin and immunoglobulins. It has also been studied for its possible immunomodulatory effects and its potential role in reducing oxidative stress and inflammation. In addition, it is considered a preventative treatment for various autoimmune diseases, especially infectious diseases. Recent research has shown that treating liver tissue with camel milk has a noticeable cumulative effect, with an increase in macrophages and lymphocytes in hepatic cells, as well as a lower granulomatous lesion with the presence of fibrosis [6]. Therefore, camel milk is considered an important aid in activating antioxidants, reducing oxidative stress, and treating acute and chronic disorders with this property [7].

Recent research has confirmed that camel milk has a unique nutritional composition that helps treat certain health problems more effectively and safely compared to other animal milks [8]. Recent studies have demonstrated that camel milk possesses significant anti‐inflammatory and antibacterial properties, as well as antioxidant properties. Camel milk also has a direct effect on reducing the effects of diabetes and acts as a vital liver protector. This is due to its high concentrations of essential vitamins and minerals, including immunoglobulins, lactoferrin, and other antioxidant compounds. Furthermore, camel milk contains very low levels of cholesterol and lactose [9, 10].

In addition, recent studies have indicated that camel milk contains important bioactive peptides and proteins that act as protective agents against oxidative stress and aid in tissue regeneration. Therefore, camel milk can be considered an important subject in biomedical and veterinary studies, particularly those related to digestive disorders, the immune system, and even liver function [7, 11, 12].

Acetylsalicylic acid, known as aspirin, is classified as a nonsteroidal anti‐inflammatory drug (NSAID), which damages the gastric mucosal membranes of the gastrointestinal tract (GIT) by increasing intestinal permeability through aspirin’s effect on the active receptor of COX‐1 cyclooxygenase and COX‐2 isoenzymes [13]. Both COX sites increase the productivity of prostaglandins in combination with arachidonic acid. Prostaglandins are linked with pain and inflammation; however, prostaglandins also stimulate the productivity of mucus, which promotes mucosal healing [14, 15]. Frequent and heavy use of aspirin can weaken the GIT barrier. Aspirin also inhibits thromboxane A2 in platelets, preventing platelet aggregation [16, 17]. Low‐dose aspirin is often used as an effective drug for the prevention of thrombotic complications. However, side effects are common, such as lesions in the lining mucosa, mild cases of indigestion, and severe or moderate gastrointestinal hemorrhage [18]. Therefore, increasing research has focused on finding natural alternatives with a protective effect against ulcers and gastrointestinal damage caused by chemical drugs like aspirin. Recent studies suggest that camel milk may be a natural agent in alleviating ulcers and inflammation of the digestive tract, thanks to its important and effective components that promote mucosal healing [19]. Recent studies have shown that camel milk has both protective and therapeutic effects against liver damage and stomach ulcers caused by chemical medications, suggesting a potential use in both preventative and veterinary medicine [20, 21].

Reactive oxygen species (ROS) and other free radicals play a major role in various degenerative disorders associated with oxidative stress [11]. Therefore, all ROS are stimulated to save cells from oxidative stress, but they cannot distinguish between infectious agents and host cells, leading to lipid peroxidation, inflammation, and tissue damage in several organs, including the GIT and liver [12, 20]. Antioxidants safeguard organisms from free radicals, but the body requires adequate quantities to oppose the destruction led by these radicals, especially from powerful agents such as aspirin. Previous research has shown that camel milk contains sufficient antioxidants, and camel milk is recommended as an important source of antioxidants to reduce and prevent oxidative damage in many body tissues [11, 22].

Despite the growing interest in modern research on the treatment of gastric ulcers and the therapeutic effect of camel milk on ulcers, its protective effect, which is crucial for aspirin‐induced gastric injuries, the scarcity, or near absence, of studies on this topic is a significant concern. This is particularly true when considering the use of important histological and biochemical techniques in rabbits as a model. Furthermore, there is a lack of recent studies on the dual effect of camel milk on all variables, injuries, and lesions of the gastric mucosa, as well as the importance of liver function indicators after prolonged aspirin use. Therefore, the current study focuses on the novel potential dual roles of camel milk in protecting both the stomach and liver simultaneously, as can be assessed through histological and biochemical evaluations in rabbits with experimentally aspirin‐induced toxicity. Therefore, this study aimed to investigate the effective preventive and potential therapeutic effects of camel milk against gastrointestinal injuries, specifically gastric and liver injuries, caused by acute aspirin toxicity in rabbits. This was achieved through histopathological and biochemical evaluation.

## 2. Materials and Method

Period preparation for the study animal: This study involved a total of 32 healthy male New Zealand white rabbits (Oryctolagus cuniculus) of 1200–1800 g and 3–5 months old. After that, the animals underwent an acclimatization period of 24 days before starting the experiment, during which they were kept collectively in a unique large cage with free access to food and tap water. Internal and external parasites were administered at weekly interval doses of albendazole, ivermectin, and emprostin as prophylactic treatments before initiation.

Chemicals and reagents: The aspirin dose was prepared two hours before use by dissolving aspirin in distilled water at a concentration of 35 mg/mL. After checking its solidity and homogeneity, its liver function biochemical parameters were determined using commercial diagnostic kits purchased from a kit used from the company Sigma/Indian.

Experimental study: Experimental animals (rabbits) were collected in suitable laboratory conditions where the temperature was controlled at 22°C–24°C, a comfortable humidity of 60%, with a 12 h light/dark cycle, and appropriate food and water were provided throughout the experimental period. The rabbits were divided into 4 equal, homogeneous random groups, each containing 8 rabbits, and subjected to the experimental period that continued for 70 days. During the experimental period, the animals were divided into the following groups.

1. Negative control group (controlling Batch S): Each animal received distilled water once daily to minimize stress‐related bias associated with oral administration.
2. Experimental Group 2 (initial processing Batch S1): Each animal received camel milk (15 mL/day).
3. Experimental Group 3 (processing Batch S2): Each animal received oral 150 mg/kg of aspirin.
4. Experimental Group 4 (processing Batch S3): Each animal received together 15 mL/day of camel milk with 150 mg/kg of body weight (BW) of the drug aspirin as medication.

All treatments were administered orally once daily for 70 consecutive days.

Sample collection: After the 70‐day experiment, blood samples were collected from the marginal ear vein of the rabbit’s ear using disposable syringes and transferred to tubes to coagulate at room temperature. The sterilization of the instruments and the puncture site was ensured for each rabbit individually. The samples were then centrifuged at 3000 rpm for 10 min to obtain serum**.**

Biochemical analysis: Serum was collected from experimental rabbits to evaluate liver functions and vital signs (clinical measurements, specifically pulse rate, temperature, respiration rate, and blood pressure, which indicate the state of essential body functions), and for biochemical tests, including ALT, AST, ALP, total protein (TP), total bilirubin (TB), and direct bilirubin (DB), according to the manufacturer’s instructions. Biochemical parameters were determined using commercial diagnostic kits according to the method described in [23].

Following euthanasia, the gastric tissue samples from all experimental animals were collected for histological examinations. These samples were transported and fixed in a 10% diluted neutral formalin buffered solution for histological processing and lesion scoring evaluation at all stages of the experiment and for all animals.

### 2.1. Detection by Macroscopic Evaluation of the Severity of Damaged Mucosa of Stomach

Before microscopic examination, a visual examination was performed. A portion of the digestive tract (stomach) was sent to the histopathology laboratory. The degree of damage resulting from the degree of hemorrhage, bleeding, and congestion of the mucosa was determined on a scale of 0–4, as per standard practice “0” refers to the type of normal mucosal/“0.5” refers to the type of hyperemia/“1” refers to the type of erosions/“2” refers to the type of severe erosions/“3” refers to the type of severe erosions and last “4” refers to the type mucosal lesions throughout the stomach, such as bleeding erosions, highly vascular congestion, and hyperemia. The ulcerative status of the gastrointestinal mucosa was assessed under a dissecting microscope using a transparent background smear at scales ranging from 1 to 2 mm. The ratio of the affected gastric mucosa zone to the overall mucosal area was marked. The proportion of aspirin‐induced damage was compared with that of the control group.

### 2.2. Detection by Histopathology in the Evaluation of the Severity of Damaged Stomach Mucosa

In the laboratory, after preserving the biopsies in a gradually concentrated ethanol solution, the samples were dehydrated, purified in xylene, infiltrated, and finally embedded in paraffin. The paraffinized blocks of tissue biopsies were sectioned at 4–5 μm of thickness using the microtome (Leica, Germany). Then, according to the company’s instructions (SYRBIO, Syria), the sections were stained with hematoxylin and eosin, and detected for morphology using a light microscope type Olympus BX51 (Tokyo, Japan) at 40 × objective magnification.

### 2.3. Histopathological Lesion Scoring

The classification riskiness of gastric mucosal damage and inflammatory cell infiltration was arranged on a severity scale from 0 to 3 as follows: A score of 0 indicates a normal appearance without detectable lesions. A score of 1 indicates focal inflammatory infiltration of the gastric mucous membrane ulceration (mild epithelial degeneration). A score of 2 indicates gastric mucosal infiltration extending into the submucosa and submucosal ulceration (moderate mucosal erosion). A score of 3 indicates a severe mucosal ulceration and hemorrhage with a gastric ulcer, leading to severe inflammatory infiltration extending into the muscle [24, 25]. However, inflammation of the gastric mucosal and submucosal layers was evaluated, and the acuteness of damage was classified on a scale of 0–3 as follows: 0, no sign; 1 indicates little; 2 indicates average; and 3 indicates severe [26, 27].

Milk collection: Milk safety was ensured by conducting MRT and CMT tests, ensuring the absence of udder lesions and mastitis [28]. Milk was collected from camels approximately 8 years old, with an average calving rate of approximately two. Thirty days after calving, the milk was milked by hand every morning after the udder was sterilized with 70% ethyl alcohol. The saved samples were in sterile, cold bags till to analyzed by the system (ultrasonic milk analyzer/Germany), and 10 mL of each sample was used for analysis according to the method [29]. Table 1 shows the detailed camel milk properties and components.

**TABLE 1:** Statistical analysis results of chemical examination.

Chemical composition	Min–max	Mean ± SD
Fat	1.32–6.9	3.3 ± 0.11
Protein	1.9–10.1	2.70 ± 0.08
Lactose	2.57–7	4.70 ± 0.08
Solid not fat	4–14.3	8.10 ± 0.14
Ash	0.15–1.1	0.70 ± 0.01
Moisture	81–96.4	88.60 ± 0.21

### 2.4. Statistical Analysis

All study data were analyzed statistically using the Statistical Analysis S (Version 9.4, SAS Institute Inc., Cary, NC, USA). Quantitative variables were expressed as mean ± standard deviation (SD), and ANOVA was used to test differences among experimental groups, followed by the LSD post hoc test. All statistical tests were two‐sided, and statistical significance was considered at *p* < 0.05 from the measured camel milk content and components [30].

### 2.5. Ethical Approval

This investigation was conducted in the anatomical facility of the University of Karbala’s College of Veterinary Medicine under reference number UOK.VET. AN. 2024.111.

## 3. Results

### 3.1. Chemical Characteristics of Camel Milk

The results indicate that the chemical composition of the camel milk used in this study is similar to the values obtained by the researchers.

### 3.2. Biochemical Examination

In the current research study, the detection of liver function enzymes by biochemical test was carried out. The study results showed a significant increase in liver function tests, and ALT levels were significantly increased in Group S2 compared with the control group (68.4 ± 24.5 vs. 62.4 ± 16.47 IU/L; one‐way ANOVA followed by LSD post hoc test, *p* = 0.03) and other indicators about liver function, TP, DB, and albumin in Batch S2 after receiving oral 150 mg/kg (76.8%, 4.8%, 6.8%, 21.1%), respectively, compared to Batch S1, as shown in Table 2. The results in Batch S3 showed an insignificant increase (*p* < 0.01) in enzymes. ALP, ALT, TP, DB, and albumin were high percentages (14.9%, 2.3%, 4.2%, 26.3%, and 20.6%), respectively, compared to Batch S1; there was an insignificant decrease in the levels of TB and AST by 0.6% and 7.1%, respectively, when compared with Batch S2.

**TABLE 2:** Level of liver function indicators in rabbit blood serum according to experimental groups.

Liver enzyme	S1	S2		S3	
Aspartate aminotransferase (AST) (IU/L)	62.1 ± 18.63^a^	55.6 ± 3.3^b^	(7.4%)	60.8 ± 28.78^a^	(0.6%)
Alanine aminotransferase (ALT) (IU/L)	62.4 ± 16.47^b^	68.4 ± 24.5^a^	(11.4%)	629 ± 20.09^b^	(2.3%)
Total protein (TP) (g/dL)	6.24 ± 0.62^b^	6.54 ± 0.35^a^	(4.8%)	6.7 ± 0.6^a^	(4.2%)
Total bilirubin (TB) (mg/dL)	0.906 ± 0.13^a^	0.868 ± 0.4^a^	(4.2%)	0.84 ± 0.07^a^	(7.1%)
Alkaline phosphatase (ALP) (IU/L)	82 ± 38.59^b^	147 ± 54.6^a^	(76.8%)	91.2 ± 60.8^b^	(14.9%)
Albumin (Alb) (g/dL)	4.48 ± 0.55^a^	5.4 ± 1.06^a^	(21.1%)	5.38 ± 1.42^a^	(20.6%)
Direct bilirubin (DB) (mg/dL)	0.231 ± 0.04^a^	0.262 ± 0.04^a^	(6.8%)	0.288 ± 0.07^a^	(26.3%)

*Note:* Different coordinating letters indicate significant variation in groups at *p* < 0.05. Values are expressed as mean ± SD.

### 3.3. Histopathological Examination

**FIGURE 1:**
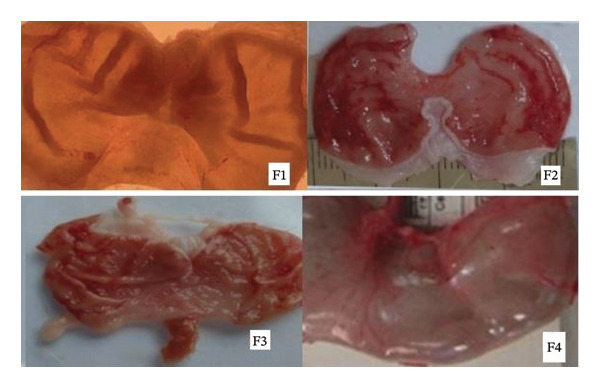
Histopathological sections of rabbit gastric tissue in different experimental groups. (F1) Negative control group (Group S) showing normal gastric mucosal architecture with intact epithelial lining. (F2) Camel milk–treated group (Group S1) demonstrating preserved mucosal integrity with minimal histopathological alterations. (F3) Aspirin‐treated group (Group S2) showing severe gastric mucosal ulceration, epithelial degeneration, inflammatory cell infiltration, congestion, and hemorrhage. (F4) Camel milk plus aspirin–treated group (Group S3) showing marked improvement in gastric tissue morphology with reduced inflammatory infiltration and partial restoration of mucosal integrity.

**FIGURE 2:**
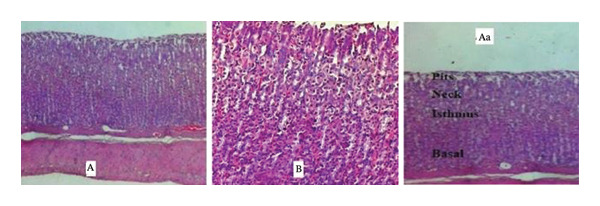
Histopathological sections of rabbit gastric mucosa from the negative control group (Group S) show normal gastric tissue architecture without detectable pathological lesions. (A) Normal gastric mucosal structure at magnification 40 ×. (Aa) Higher magnification view (100 ×) showing intact epithelial lining and normal gastric glands. (B) Normal histological appearance of gastric tissue demonstrating preserved mucosal integrity and absence of inflammatory or degenerative alterations at magnification 100 ×. Hematoxylin and eosin (H&E) stain, 40× and 100× magnification.

On microscopic examination, no significant (*p* > 0.05) histopathological variations were reported by the microscopically examined mucosal or submucosal injury of both first and second experiments in the stomach mucosa. However, hemorrhagic patches (arrows) in Batch S1 (Group 2) appear microscopically, and the peptic sores were mostly extensive and stringy, with an increased danger of microscopic. The incidence of infection was significantly higher (*p* < 0.05) in the S1–S2 cohort compared to the control group in the mucosa and submucosa. However, the S2 group showed greater macroscopic mucosal damage compared to S1 (*p* < 0.05), as shown in Table 3 and Figure 1 (F1, F2, and F3). In addition, there appears to be no lesion in the Batch S–S1 on histological evaluation Figure 2 (A, Aa, B).

**TABLE 3:** Camel milk influence on histological parameters in rabbits given aspirin.

Treatments	S	S1	S2	S3
Mucosal damage (macroscopic)	0.31 ± 0.11^b^	0.00 ± 0.00^b^	2.26 ± 0.51^a^	1.52 ± 0.55^ab^
Mucosal damage (histological)	0.19 ± 0.67^b^	0.16 ± 0.66^a^	2.08 ± 0.81^a^	087 ± 0.54^ab^
Inflammation (score)	0.48 ± 0.27^b^	0.33 ± 0.16^b^	2.41 ± 0.56^a^	1.22 ± 0.42^ab^

*Note:* Different coordinating letters indicate significant variation in groups at *p* < 0.05. Values are expressed as mean ± SD.

As shown by Batch S1, epithelial cell necrosis including vacuolar formation in the pits and neck areas (arrows) with bleeding and some infiltration of inflammatory cells in the affected areas was observed at the arrowheads, whereas in the second Batch S2, there was mild focal necrosis of the epithelial cells as shown by the arrows, along with inflammatory cells in the fossa region as shown by the arrowheads neck and isthmus areas, limited to the ciliary, mucosa.

**FIGURE 3:**
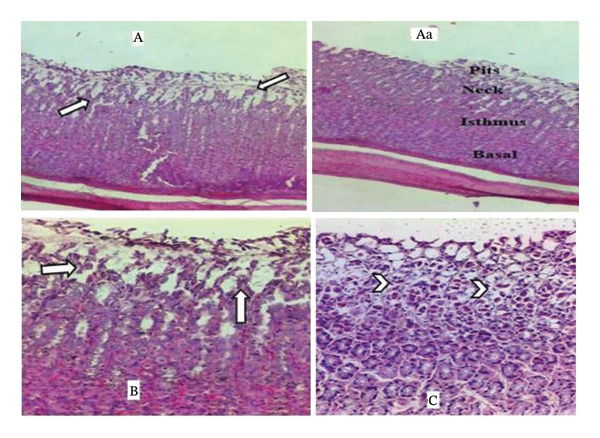
Histopathological sections of rabbit liver tissue in different experimental groups. (A) Negative control group (Group S) showing normal hepatic architecture with normal hepatocytes and central vein structure. (Aa) The camel milk–treated group (Group S1) demonstrated nearly normal hepatic tissue with mild or absent histopathological alterations. (B) The aspirin‐treated group (Group S2) showed severe hepatocellular degeneration, sinusoidal dilatation, inflammatory cell infiltration, vascular congestion, and necrotic changes. (C) The camel milk plus aspirin–treated group (Group S3) showed marked improvement in hepatic tissue morphology with reduced cellular degeneration and inflammatory infiltration compared with the aspirin‐treated group. Hematoxylin and eosin (H&E) stain “A, Aa used x40 while B, C used x100.”

**FIGURE 4:**
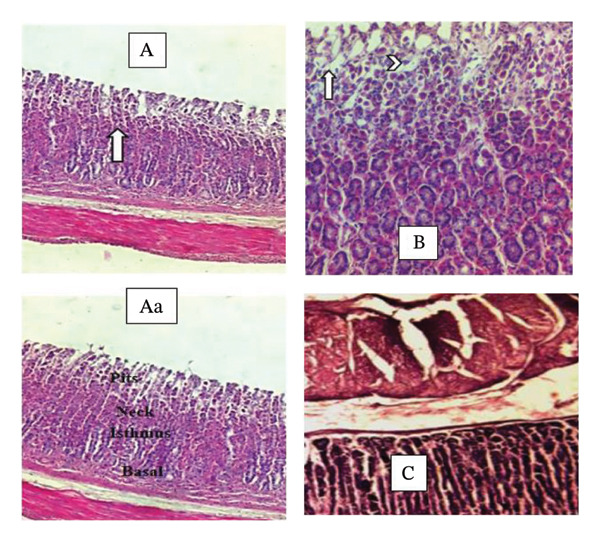
Histopathological sections demonstrating gastric and hepatic tissue alterations among different experimental groups. (A) Negative control group (Group S) showing normal tissue architecture without detectable pathological lesions. (Aa) The camel milk–treated group (Group S1) demonstrated preserved tissue morphology with minimal histopathological alterations. (B) The aspirin‐treated group (Group S2) showed severe degenerative and inflammatory lesions characterized by epithelial degeneration, cellular infiltration, vascular congestion, and tissue damage. (C) The camel milk plus aspirin–treated group (Group S3) showed marked histopathological improvement with reduced inflammatory changes and restoration of normal tissue architecture compared with the aspirin‐treated group. Hematoxylin and eosin (H&E) stain “A Aa used x40 while B, C used x100.”

Batch S2 showed deep ulcers reaching and attached to the layer of areolar connective tissue lying beneath a mucous membrane, while the third Batch S3 did not appear as shown in Table 3, Figure 3 (A, Aa, B, C), and Figure 4 (A, B, C). The mucosal and submucosal inflammation of the lesion was missing in Batches S and S1, as shown in Figure 2 (A, B). Batch S2 showed highly severe inflammation, but Batch S3 presented reasonable inflammation to significantly less (*p* < 0.05) than that of second Batch S2 (A, B, and C) 5, as shown in Table 3 and Figure 3 (A–B).

### 3.4. BW Condition

The current study, after weekly weight monitoring of rabbits for 70 days during the experimental period, as shown in Table 4, revealed that Groups S1 and S3 showed stable and improved BW compared to Group S2, which showed a slight decrease in weight compared to the control group. However, the differences between the experimental groups were not statistically significant (*p* > 0.05).

**TABLE 4:** Changes in body weight of rabbits by group during the experimental period.

Groups	Weight before the experiment (g)	Weight after the experiment	Changes in weight (g)
Group S (control)	1450 ± 85	1818 ± 96	368 ± 28
Group S1	1443 ± 91	1889 ± 103	446 ± 32^∗^
Group S2	1460 ± 89	1618 ± 93	158 ± 27^∗∗^
Group S3	1452 ± 84	1760 ± 98	308 ± 28^#^

*Note:* (*p* < 0.05) Values are expressed as mean ± SD (*n* = 8).

^∗^Significant increase.

^∗∗^Significant decrease.

^#^Significant improvement.

## 4. Discussion

The results of the current study indicate that camel milk has a protective effect due to its high content of antioxidants and biologically important compounds [31]. Active compounds such as lactoferrin, vitamins C and E, and immunoglobulins play a vital role, with important minerals like zinc and selenium contributing to their biological activity as antioxidant enzymes [9, 32]. Camel milk components help to reduce the inflammatory reactions resulting from oxidative stress associated with tissue damage caused by aspirin. Camel milk components help to reduce inflammatory reactions resulting from oxidative stress and tissue damage induced by aspirin [33]. Aspirin, as a drug, is known to have biological effects on a major health problem in the digestive system, causing significant malformations and potentially toxic gastric and duodenal ulcers. It poses a risk to public health by physiologically inhibiting prostaglandin synthesis, increasing oxidative stress, causing vascular congestion, and promoting the infiltration of inflammatory cells, leading to bleeding and damage in the digestive tract [15, 17] and life‐threatening conditions in both acute and long‐term exposures [34]. Therefore, camel milk reduced the damage to the stomachs of rabbits treated with it due to its high antioxidant content, which protected stomach cells from damage. This was confirmed by a recent study [35], consistent with the results of the current study, which showed that camel milk possesses potent anti‐inflammatory and anti‐ulcer properties in the stomach. These properties are achieved by regulating ROS and supporting the healing of the gastric mucosa [8, 19]. Furthermore, the proteins and bioactive peptides in camel milk aid in the healing process and regeneration of epithelial cells, leading to a reduction in necrosis of gastric tissue cells. This aligns with the findings of previous studies that described camel milk as a key agent in treating oxidative gastric tissue damage [35, 36].

The effect of camel milk on improving biochemical indicators and liver functions is also linked to the effects of aspirin. The antioxidant properties of camel milk help reduce the effects of lipid peroxidation and oxidative damage to liver cells, thus maintaining liver function and increasing its enzyme balance, consistent with the findings of [36, 37]. Furthermore, recent studies indicate that camel milk enhances immune responses and reduces the production of harmful inflammatory cytokines. This, in turn, reduces inflammation in the tissue and promotes rapid recovery from aspirin‐induced damage in rabbits [26, 27, 38].

The results of histological examination, particularly of the pathological tissue, confirmed the effect of camel milk on gastric ulcer in rabbits, reducing inflammation and cellular infiltration, and decreasing the severity of inflammation and mucosal lesions in the rabbits treated with camel milk. This is attributed to the combined protective effect of the vitamins, minerals, and proteins in camel milk, which act as natural antioxidants and immune modulators [39, 40].

Additionally, when compared to the nutritional content, some non–camel milks contain less sugar, protein, and even fats like cholesterol than camel milk, and also include the top levels of support nutrients, such as minerals, with vitamins, especially vitamin C, due to the environmental and nutritional characteristics of camels [39–41]. Furthermore, consuming camel milk increases the absorption of calcium, which is essential for bone formation, making it effective in treating calcium metabolism disorders [42]. That is why camel milk has been reported to enhance immune responses and exert protective effects against various pathological conditions, such as peptic ulcers and microbial infections [43, 44]; therefore, camel milk is used in different domains [1, 3]. Economically, camel milk has been introduced as a staple food through direct consumption as pasteurized liquid milk, which is often the most widely consumed in the world due to low allergens [45, 46], as previously mentioned. Dairy manufacturers have also discovered that camel milk assists in promoting the alkalinity and elevation perfection of the skeletal system in the pH of the blood system, which reduces and prevents intestinal bleeding and peptic ulcers [44, 47]. Additionally, the beneficial medium‐chain fatty acids, such as capric and caprylic acid, in camel milk help combat diseases and pathogens. It helps to improve and strengthen immunity and is a powerful antioxidant [48]. Generally, the findings of the current study demonstrated the protective and therapeutic effect of camel milk against aspirin‐induced gastric and liver damage. Therefore, further studies are recommended, particularly molecular and immunological studies to support the mechanisms by which camel milk affects the biological body. However, to assess oxidative stress markers and antioxidant enzyme activities, such as malondialdehyde (MDA) and reduced glutathione (GSH), to better clarify the molecular mechanisms underlying the protective effects of camel milk.

## 5. Conclusions

The current study concluded that camel milk has beneficial properties, including strong antioxidant activity, immune‐enhancing effects, potential to treat and prevent inflammation, and the ability to improve the healing of irritated gastric mucosa induced by aspirin. However, the positive findings of this study on camel milk may still preclude a full investigation into the precise mechanisms and therapeutic effects of camel milk against gastric damage and inflammation. A deeper study on microbiological assessment could also be conducted, and research must be conducted on the therapeutic importance of camel milk on GIT disturbance, as well as its immune‐boosting and impact on body health through the half‐life of camel milk treatment.

## Funding

No funding has been received for this research.

## Disclosure

All authors have read and approved the final version of the manuscript.

## Conflicts of Interest

The authors declare no conflicts of interest.

## Data Availability

The authors confirm that the data supporting the findings of this study are available from the corresponding author upon reasonable request. The corresponding author had full access to all study data and takes complete responsibility for the integrity of the data and the accuracy of the data analysis.

## References

[1] Al-Aalim A. , Sheet O. H. , Al-Jumaa Z. M. , and Hamad M. A. , Molecular Detection of Mycoplasma spp. From Camel’s Milk, 2023, 10.33899/IJVS.2022.134635.2388.

[2] Sheet O. H. , Al-Aalim A. M. , Al-Jumaa Z. M. , and Al-Sanjary R. A. , Molecular Detection of *Escherichia coli* Isolated From Camel Milk in Nineveh Governorate, Iraqi Journal of Veterinary Sciences. (April 2024) 38, no. 2, 329–333, 10.33899/ijvs.2023.141222.3106.

[3] Semsmia R. , Abed A. L.-R. T. , and Al-Daker Ÿ. B. , Impact of Parity, Stage of Lactation, and Subclinical Mastitis on the Concentration of Vitamin C in Shami Camel Milk, Iraqi Journal of Veterinary Sciences. (2022) 36, no. 4, 847–851, 10.33899/ijvs.2022.132279.2078.

[4] Krishnankutty R. , Iskandarani A. , Therachiyil L. et al., Anticancer Activity of Camel Milk via Induction of Autophagic Death in Human Colorectal and Breast Cancer Cells, Asian Pacific Journal of Cancer Prevention: APJCP.(2018) 19, no. 12, 3501–3509, 10.31557/apjcp.2018.19.12.3501. 30583676 PMC6428541

[5] Galali Y. and Al-Dmoor H. M. , Miraculous Properties of Camel Milk and Perspective of Modern Science, Journal of Family Medicine and Disease Prevention. (2019) 5, no. 1, 1–7.

[6] Omer A. G. and Dol Ateye M. , Analysis of Physical and Microbiological Quality of Raw Camel Milk in the Somali Regional State of Ethiopia, Online Journal of Animal and Feed Research. (2022) 12, no. 6, 372–378.

[7] Chethouna F. , Boudjenah S. H. , Beldi N. , and Siboukeur O. , Comparative Study of the Physico-Chemical and Microbiological Characteristics of Raw and Pasteurized Camel Milk, Emirates Journal of Food and Agriculture. (2022) 34, no. 10, 850–858.

[8] Bereda G. , Uthirapathy S. , and Ahamad J. , Camel Milk as a Functional Food: Nutritional Composition, Health‐Promoting Benefits, and Safety Considerations, Food Science and Nutrition. (March 2026) 14, no. 3, 10.1002/fsn3.71638. PMC13093688 42016254

[9] Behrouz S. , Saadat S. , Memarzia A. , Sarir H. , Folkerts G. , and Boskabady M. H. , The Antioxidant, Anti-Inflammatory and Immunomodulatory Effects of Camel Milk, Frontiers in Immunology. (April 2022) 13, 10.3389/fimmu.2022.855342. PMC9039309 35493477

[10] Hassan S. M. , Tola Y. B. , Forsido S. F. , Arimi J. , Teka T. A. , and Urugo M. M. , Nutritional Composition, Bioactive Compounds, Functional Properties, and Processing Technologies of Camel Milk and Its Value-Added Products: A Review, Applied Food Research. (March 2026) 28, no. 1, 10.1016/j.afres.2026.101949.

[11] Saleem H. D. , Al-Obaidi A. H. , and Al-Tmemy W. B. , Histopathological Changes due to Toxic Effect of Aflatoxin B1 on Liver, Kidney and Therapeutic/Preventive Role of Camel Milk, Biochemical and Cellular Archives. (April 2021) 21, no. 1.

[12] Kocyigit E. , Abdurakhmanov R. , and Kocyigit B. F. , Potential Role of Camel, Mare Milk, and Their Products in Inflammatory Rheumatic Diseases, Rheumatology International. (March 2024) 44, no. 3, 425–434, 10.1007/s00296-023-05516-x. 38183445 PMC10867071

[13] Briggs N. G. , Brennan K. M. , Funnell B. J. , Nicholls G. T. , and Schoonmaker J. P. , Use of Aspirin to Intentionally Induce Gastrointestinal Tract Barrier Dysfunction in Feedlot Cattle, Journal of Animal Science. (Septembe 2020) 98, no. 9, 10.1093/jas/skaa264. PMC7497817 32815992

[14] Takeuchi K. and Amagase K. , Roles of Cyclooxygenase, Prostaglandin E2 and EP Receptors in Mucosal Protection and Ulcer Healing in the Gastrointestinal Tract, Current Pharmaceutical Design. (May 2018) 24, no. 18, 2002–2011, 10.2174/1381612824666180629111227. 29956615

[15] Yahya T. and Mousa Y. J. , Pharmacodynamic and Pharmacokinetic Comparison Between Selective and Non-Selective COX-2 Inhibitors in Mice, Journal of Applied Veterinary Sciences. (April 2024) 9, no. 2, 99–105.

[16] Patrick J. , Dillaha L. , Armas D. , and Sessa W. C. , A Randomized Trial to Assess the Pharmacodynamics and Pharmacokinetics of a Single Dose of an Extended-Release Aspirin Formulation, Postgraduate Medicine. (November 2015) 127, no. 6], 573–580, 10.1080/00325481.2015.1050341. 25998572

[17] Cardile S. , Martinelli M. , Barabino A. et al., Italian Survey on Non-Steroidal Anti-Inflammatory Drugs and Gastrointestinal Bleeding in Children, World Journal of Gastroenterology. (February 2016) 22, no. 5. 10.3748/wjg.v22.i5.1877 PMC4724619 26855547

[18] Gralnek I. M. , Neeman Z. , and Strate L. L. , Acute Lower Gastrointestinal Bleeding, New England Journal of Medicine. (March 2017) 376, no. 11, 1054–1063. 28296600 10.1056/NEJMcp1603455

[19] AL-Lbban A. M. , The Role of Camel Milk as a Protective Factor on Rats Infected With Indomethacin-Induced Gastric Ulcer, Food Science and Technology. (August 2024) 44, 10.5327/fst.00280.

[20] Ahmed A. A. , Saad N. M. , Wahba N. M. , and Sayed R. G. , Nutritional Value and Antioxidant Activity of Camel’s Milk, Journal of Advanced Veterinary Research. (October 2018) 8, no. 4, 90–94.

[21] Behrouz S. , Mohammadi M. , Sarir H. , and Boskabady M. H. , The Effects of Camel Milk in Systemic Inflammation and Oxidative Stress of Cigarette Smoke-Induced Chronic Obstructive Pulmonary Disease Model in Rat, Frontiers in Veterinary Science. (December 2024) 11, 10.3389/fvets.2024.1464432. PMC11673985 39735585

[22] Ouda Y. W. , Kadhim K. F. , and Amer A. M. , Study of Some Toxic Metals in Parts From Catfish [*Silurus triostegus*] in Shatt al-Arab River, Iraqi Journal of Veterinary Sciences. (2023) 37, no. 2, 459–467, 10.33899/ijvs.2022.135004.2435.

[23] Didkowska A. , Klich D. , Anusz K. et al., Determination of Hematological and Biochemical Values Blood Parameters for European Bison (*Bison bonasus*), PLoS One. (May 2024) 19, no. 5, 10.1371/journal.pone.0303457. PMC11095690 38748744

[24] Otsuka T. , Sugimoto M. , Ban H. et al., Severity of Gastric Mucosal Atrophy Affects the Healing Speed of Post-Endoscopic Submucosal Dissection Ulcers, World Journal of Gastrointestinal Endoscopy. (May 2018) 10, no. 5, 83–92, 10.4253/wjge.v10.i5.83. 29774087 PMC5955726

[25] Al-Sabawy H. B. , Rahawy A. M. , and Al-Mahmood S. S. , Standard Techniques for Formalin-Fixed Paraffin-Embedded Tissue: A Pathologist’s Perspective Iraqi, Journal of Veterinary Science. (2021) 35, no. I-III, 127–135.

[26] Di Bella S. , Luzzati R. , Principe L. et al., Aspirin and Infection: A Narrative Review, Biomedicines. (January 2022) 10, no. 2, 10.3390/biomedicines10020263. PMC8868581 35203473

[27] Alqahtani S. , The Antiplatelet Activity of Camel Milk in Healthy and Aluminum Chloride-Intoxicated Rats, Saudi Journal of Biological Sciences. (August 2022) 29, no. 8, 10.1016/j.sjbs.2022.103369. PMC9287608 35855769

[28] Stanek P. , Żółkiewski P. , and Januś E. , A Review on Mastitis in Dairy Cow’s Research: Current Status and Future Perspectives, Agriculture. (August 2024) 14, no. 8, 10.3390/agriculture14081292.

[29] Tamimi N. S. and Al-Shihani S. N. , Assessment of the Milk Components of Iraqi Dromedary Camels, Basrah Journal of Veterinary Research. (2020) 19, no. 3, 80‐89–89, 10.23975/bjvetr.2020.174099.

[30] Saleem H. D. and Al-Samarai F. R. , Prevalence of Theileriid Equip in Horses in Central Iraq Determined by Microscopy and PCR, Prevalence.(2018) 22, no. 4, 273–280.

[31] Musa K. H. , Hamad E. M. , and Elnour A. A. , Camel Milk and Oxidative Stress: Therapeutic Potential Against Metabolic Syndrome Diseases, Journal of Agriculture and Food Research. (Mar 2025) 19, 10.1016/j.jafr.2025.101682.

[32] Khaliq A. , Mishra A. K. , Niroula A. , Baba W. N. , Shaukat M. N. , and Rabbani A. , An Updated Comprehensive Review of Camel Milk: Composition, Therapeutic Properties, and Industrial Applications, Food Bioscience. (December 2024) 62, 10.1016/j.fbio.2024.105531.

[33] Li Y. , Ma Q. , Li M. et al., Non-Bovine Milk as Functional Foods With Focus on Their Antioxidant and Anti-Inflammatory Bioactivities, Antioxidants. (June 2025) 14, no. 7, 10.3390/antiox14070801. PMC12291644 40722905

[34] Abed H. R. and Sahib A. M. , Protective Effect of Raw Goat Milk Against Aspirin Induced Gastric Ulcer in Rats, Revista Electronica de Veterinaria. (2022) 382–392.

[35] Al-Tamimi J. , Alhazza I. M. , Ebaid H. et al., Macrophage Plasticity Enhanced by Camel Milk Peptide Attributes in Wound Healing in Diabetic Rats, Journal of King Saud University Science. (March 2024) 36, no. 3, 10.1016/j.jksus.2023.103088.

[36] Mandot S. and Kumar Meena K. , Camel Milk-Derived Bioactive Peptides: A Comprehensive Review of Their Production Methods and Bioactive Properties, African Journal of Biological Sciences. (2024) 6, no. Si3, 3449–3462.

[37] Simon T. G. , Duberg A. S. , Aleman S. , Chung R. T. , Chan A. T. , and Ludvigsson J. F. , Association of Aspirin With Hepatocellular Carcinoma and Liver-Related Mortality, New England Journal of Medicine. (March 2020) 382, no. 11, 1018–1028, 10.1056/nejmoa1912035. 32160663 PMC7317648

[38] Abou-Soliman N. H. , Abd-Rabou H. S. , Awad S. , and Ibrahim A. A. , Impact of Thermal Treatment on the Quality, Total Antioxidant and Antibacterial Properties of Fermented Camel Milk, Scientific Reports. (March 2025) 15, no. 1, 10.1038/s41598-025-91548-1. PMC11903780 40074796

[39] Mohammadabad T. , Camel Milk as an Amazing Remedy for Health Complications: A Review, Basrah Journal of Agricultural Sciences. (October 2020) 33, no. 2, 125–137.

[40] Al-Rubaye H. , Atiyah A. J. , and Huang S. , Comparative Study of Aflatoxin M1 Carry-Over From Feed to Raw Milk in Cow, Buffalo, Sheep, and Goats in Different Areas of Boghdad Province, Iraqi Journal of Veterinary Medicine. (December 2023) 47, no. 2, 88–93, 10.30539/8svc0t05.

[41] Shati A. A. , Al-Taee H. S. , and Saleem H. D. , Ser-Surveying of Caprine Q-Fever (*Coxiella burnetii*) in Milk, REDVET-Revista Electrónica de Veterinaria. (August 2022) 23, no. 3.

[42] Fayyad A. F. and Alzuheir I. M. , Pathological Detection of Nutritional Muscular Dystrophy in Dromedary Camel Calves in Palestine, Iraqi Journal of Veterinary Sciences. (2023) 37, no. 4, 943–947, 10.33899/ijvs.2023.138289.2784.

[43] Arain M. A. , Salman H. M. , Ali M. et al., A Review on Camel Milk Composition, Techno-Functional Properties and Processing Constraints, Food science of animal resources. (July 2024) 44, no. 4, 739–757, 10.5851/kosfa.2023.e18. 38974725 PMC11222694

[44] Abd El-Aziz M. , Kassem J. M. , Aasem F. M. , and Abbas H. M. , Physicochemical Properties and Health Benefits of Camel Milk and Its Applications in Dairy Products: A Review, Egyptian Journal of Chemistry. (May 2022) 65, no. 5, 101–118.

[45] Arrichiello A. , Auriemma G. , and Sarubbi F. , Comparison of Nutritional Value of Different Ruminant Milks in Human Nutrition, International Journal of Functional Nutrition. (June 2022) 3, no. 4, 10.3892/ijfn.2022.28.

[46] Almashhadany D. A. , Mohammed H. I. , Zaki A. M. , and Hassan R. R. , Reliable and Highly Specific Techniques for the Detection of *Brucella* spp. Antibodies in Camel Milk, Iraqi Journal of Veterinary Sciences. (2023) 37, no. 4, 795–800, 10.33899/ijvs.2023.137092.2637.

[47] Alhassani W. E. , Camel Milk: Nutritional Composition, Therapeutic Properties, and Benefits for Human Health, Open Veterinary Journal. (December 2024) 14, no. 12, 10.5455/ovj.2024.v14.i12.2. PMC11799641 39927355

[48] Abubakar A. L. , Dandare A. , Dandare S. U. , Rabiu S. , Ibrahim A. S. , and Armaya’u S. , Effect of Camel Milk Supplementation in Management of Gastric Ulcer, Applied Medical Research. (2018) 4, no. 1, 12–17, 10.5455/amr.20180724045336.

